# Speed at the edge of stability: a recovery-limited model of metabolic flux in the cheetah

**DOI:** 10.1093/conphys/coag087

**Published:** 2026-11-10

**Authors:** Adrian S W Tordiffe, Tertius Kohn

**Affiliations:** Greens Zoological Rescue and Rehabilitation Centre, SSO, A5, Village Moti Khavdi, District Jamnagar, Gujarat 361142, India; Department of Paraclinical Sciences, Faculty of Veterinary Science, University of Pretoria, Soutpan Road, Onderstepoort 0110, Gauteng, South Africa; Department of Paraclinical Sciences, Faculty of Veterinary Science, University of Pretoria, Soutpan Road, Onderstepoort 0110, Gauteng, South Africa; Department of Medical Biosciences, Faculty of Natural Sciences, University of the Western Cape, Robert Sobukwe Road, Bellville 7535, South Africa

**Keywords:** Acid–base balance, *Acinonyx jubatus*, exercise physiology, feeding ecology, metabolomics, phosphocreatine, recovery physiology, wildlife conservation

## Abstract

The cheetah (*Acinonyx jubatus*) achieves unmatched terrestrial speed through anatomical and muscular specialization for explosive power. Its type IIX glycolytic muscle phenotype, phosphocreatine (PCr) buffering and rapid glycolysis supply adenosine triphosphate at exceptionally high rates. This design enables acceleration but carries a metabolic cost: PCr is depleted, inorganic phosphate (Pi) and proton balance are disturbed, lactate is redistributed, and glucose and glycogen are used. We propose that feeding ecology may add to this cost. After a successful hunt, a substantial carcass meal imposes a second load through gastric acid secretion, the alkaline tide, pancreatic and biliary bicarbonate flux, bile acid signalling, and amino acid and lipid processing. Recovery starts when the chase ends, but its components resolve at different rates. PCr resynthesis and lactate transport may begin within minutes, whereas acid–base and electrolyte normalization, glycogen replenishment and intermediary-metabolite clearance may take longer. Feeding may therefore begin while slower recovery processes remain active, allowing the two loads to overlap. The model predicts that shorter hunt-to-feed or inter-meal intervals will increase the magnitude, duration or cumulative carry-over of acid–base, electrolyte and metabolomic perturbations. Direct cheetah evidence includes the fast-glycolytic muscle phenotype, high spot urinary creatinine concentrations, abundant urinary organic acids and altered acylcarnitine profiles. The timing and interaction of sprint recovery, digestion and metabolite clearance remain unmeasured. This Perspective integrates the adaptations that permit exceptional speed with their potential metabolic costs and provides a testable framework for evaluating how feeding ecology and managed feeding schedules influence recovery.

## Abbreviations

ADP, adenosine diphosphate
ATP, adenosine triphosphate
CoA, coenzyme A
GLUT4, glucose transporter type 4
NAD+, oxidized nicotinamide adenine dinucleotide
PCr, phosphocreatine
Pi, inorganic phosphate.

## Introduction

The cheetah (*Acinonyx jubatus*) is an extreme specialist for short-duration, high-intensity locomotion. Its acceleration and sprint speed are supported by a light skeleton, elongated limbs and a highly specialized musculoskeletal system. Direct muscle studies also show a strong predominance of type IIX glycolytic fibres and high glycolytic enzyme activity (Williams *et al.*, 1997; Hudson *et al.*, 2011*a*; Hudson *et al.*, 2011*b*; Hudson *et al.*, 2012; Wilson *et al.*, 2013; Meachen *et al.*, 2018; Kohn *et al.*, 2024). Together, these anatomical and metabolic adaptations allow adenosine triphosphate (ATP) to be supplied at exceptional rates and make explosive acceleration possible, but they favour power over efficiency. This Perspective examines both sides of that specialization: the physiology that enables extreme speed and the metabolic cost that follows.

The central hypothesis of this Perspective is that the same adaptations that permit exceptional sprinting also create a substantial metabolic cost, and that the cheetah’s feeding ecology can add to that cost. During the chase, rapid phosphocreatine (PCr) turnover and glycolysis deplete PCr, increase inorganic phosphate (Pi) and proton load, redistribute lactate, and draw on glucose and glycogen. This is the first metabolic challenge. After a successful hunt, consumption of the carcass creates a second. Gastric acid secretion, the alkaline tide, pancreatic and biliary bicarbonate flux, bile acid signalling, and the processing of a large protein and lipid load impose a separate gastrointestinal, acid–base and metabolic burden. Feeding is therefore not simply restorative; it may amplify the metabolic cost of sprinting by adding a new physiological load before all slower recovery processes have resolved.

Recovery begins as soon as sprinting stops, but the relevant processes do not finish together. PCr resynthesis and lactate transport may proceed quickly, whereas acid–base and electrolyte balance, glycogen stores and downstream metabolite processing recover along different and potentially longer trajectories. The key question is therefore not whether the cheetah is globally recovered before eating, but which variables remain displaced when feeding begins and whether the second load amplifies or prolongs the metabolic cost of the chase.

In this paper, high flux refers to ATP turnover or substrate processing that is rapid relative to downstream oxidative, conjugation or excretory capacity. A recovery-limited state occurs when the next physiological input begins before at least one relevant variable has returned towards its pre-event trajectory, producing measurable carry-over. Metabolic stability is the capacity to contain these disturbances and return the relevant variables towards baseline without progressive displacement across successive events.

The hypothesis makes clear predictions. Changing the interval between exertion and feeding should alter the magnitude or duration of the combined response if exercise recovery and digestion interact. Shortening the interval between meals should produce cumulative carry-over if slower post-prandial processes remain unresolved. The model would be weakened if candidate variables consistently normalize before feeding, or if altering these intervals has no effect. Direct cheetah evidence currently supports the muscle phenotype and several metabolomic observations, but the proposed temporal interactions remain largely untested. Table 1 separates those observations from comparative inference and from the predictions of the model.

**Table 1:** Current evidentiary status and critical tests of the proposed recovery-limited model of cheetah metabolism

**Model component**	**Direct cheetah evidence**	**Comparative inference or unresolved point**	**Discriminating test/result that would weaken the model**
Sprint phenotype	Wild cheetahs perform brief high-speed pursuits (Wilson *et al.*, 2013). Skeletal muscle is rich in type IIX glycolytic fibres and shows high glycolytic enzyme activities relative to several oxidative enzyme activities (Williams *et al.*, 1997; Kohn *et al.*, 2024).	PCr and glycolysis are expected to dominate early ATP supply, but *in vivo* ATP partitioning and the variable that terminates a hunt are unknown.	Measure muscle energetics through controlled sprinting. Little PCr/glycolytic perturbation, or no relation between candidate variables and performance, would weaken this component.
Lactate, proton and Pi handling	No chase-time measurements of muscle lactate, pH, Pi or monocarboxylate transporter capacity are available.	Mammalian evidence distinguishes lactate production and shuttling from proton- and Pi-associated contractile impairment.	Serial lactate and blood gases plus direct muscle measurements. Trivial perturbation or return of the relevant variables towards baseline before feeding would weaken an overlap mechanism.
PCr and urinary creatinine	Spot urine creatinine was 12.9–115.4 mmol L^−1^ (mean 48.64 mmol L⁻¹) in captive cheetahs; no PCr kinetics or absolute daily excretion were measured (Tordiffe *et al.*, 2017*a*).	Spot concentration reflects urine dilution, muscle mass, creatine-pool size and renal handling; it does not demonstrate acute PCr turnover.	Measure total creatine, exercise-related PCr depletion/resynthesis and standardized absolute excretion. Unexceptional values would weaken the proposed emphasis on this system.
Timing of feeding relative to recovery	Feeding after successful hunts is documented (Caro, 1994), but chase-to-first-ingestion latency and physiological recovery state at first ingestion are poorly quantified.	PCr, lactate, pH, glucose, glycogen and metabolite profiles recover on different timescales that may be uniquely modified in cheetahs.	Pair behavioural timing with serial physiology and manipulate feeding delay. Return of the relevant variables towards baseline before first ingestion, or no interval-dependent response would weaken the model.
Post-prandial acid–base and electrolyte responses	No serial cheetah data define the alkaline tide, bicarbonate flux or electrolyte changes after a large meal.	Mammalian gastric HCl secretion, pancreatic and biliary bicarbonate, and renal and gastrointestinal exchange predict post-prandial pH, HCO₃^**−**^, Cl^**−**^ and K^+^ shifts.	Serial blood gases and plasma/urine electrolytes after feeding. Absence of reproducible responses or exercise-feeding interaction would weaken this component.
Organic acids and glycine conjugation	Captive cheetah urine contains abundant aromatic acids and glycine conjugates (Tordiffe *et al.*, 2017).	High pathway flux, CoA sequestration and conditional glycine limitation are proposed; urine does not localize the conjugating tissue.	Timed plasma/urine profiles and glycine or isotope studies. Clearance independent of glycine status and recovery interval would weaken the proposed bottleneck.
Acylcarnitines and oxidative processing	Thesis data describe captive–free-ranging differences in serum acylcarnitines, but samples were not timed to exercise or feeding (Tordiffe, 2017).	Acylcarnitines can reflect incomplete fatty-acid oxidation or acyl-group buffering, but are context dependent.	Serial profiles across fasting, exercise and feeding. Lack of state dependence or relation to recovery would weaken the overflow interpretation.
Managed-care recovery and disease	Gastrointestinal disease and metabolic abnormalities occur in managed cheetahs (Tordiffe, 2026), but a causal effect of feeding interval has not been demonstrated.	Short intervals may produce carry-over; diet composition, food handling, stress, activity and institutional effects are alternative explanations.	Manipulate interval while holding diet, intake, body condition and activity constant. No interval effect after controlling confounders would argue against this link.

Direct evidence refers to measurements or observations made in cheetahs. Comparative inference refers to mechanisms established in other mammals but not yet demonstrated in cheetahs. Critical tests identify outcomes that would support or weaken each model component*.*

### The adaptations and metabolic cost of high-speed pursuit

Evidence from humans and other mammals provides the working framework for the immediate energy supply during a cheetah sprint. The first seconds of maximal contraction are supported by the small ATP pool already present in muscle and by the PCr system. Through the creatine kinase reaction, PCr donates phosphate to adenosine diphosphate (ADP) and briefly maintains ATP concentration while PCr stores fall (Baker *et al.*, 2010; Hargreaves and Spriet, 2020). This is the highest rate of ATP supply available to mammalian skeletal muscle and is well suited to the initial acceleration phase of a cheetah chase.

As PCr availability declines, glycogenolysis and glycolysis increase their contribution to ATP supply and help sustain high power output. Conversion of pyruvate to lactate regenerates oxidized nicotinamide adenine dinucleotide (NAD+) and allows glycolysis to continue. Lactate formation should not be treated as the cause of intracellular acidosis. The fall in muscle pH reflects the net balance of proton-producing and proton-consuming reactions, with rapid ATP hydrolysis providing an important proton source. Pi and, under some conditions, increased proton load can impair cross-bridge cycling, calcium handling and shortening velocity (Allen *et al.*, 2008; Brooks, 2018; Brooks *et al.*, 2023; Debold and Westerblad, 2024). Lactate and H^+^ are co-transported from contracting fibres through proton-linked monocarboxylate transporters. Lactate can then be oxidized by other tissues or used for gluconeogenesis.

Intense exercise also increases skeletal muscle glucose uptake through translocation of glucose transporter type 4 (GLUT4) (Richter, 2021). Muscle glycogen and circulating glucose may therefore contribute to both performance and recovery, although complete glycogen depletion is unlikely during a single short chase.

Cheetah-specific evidence strongly supports a rate-oriented muscle phenotype. Cheetah skeletal muscle contains a high proportion of type IIX glycolytic fibres, high lactate dehydrogenase activity and comparatively lower activities of oxidative enzymes such as citrate synthase and 3-hydroxyacyl-CoA dehydrogenase (Williams *et al.*, 1997; Kohn *et al.*, 2024). This profile favours rapid glycolytic ATP production over sustained oxidative work.

Taken together, these features support a model in which PCr buffering and glycolysis provide rapid, overlapping ATP supply during a chase. The sprint is expected to alter PCr availability, Pi and proton balance, lactate distribution, glucose use and glycogen stores. These changes begin to recover as soon as running stops and provide the first metabolic perturbation in the proposed sequence.

The relative contribution and timing of each energy system have not been measured during natural cheetah chases, and the proximate cause of hunt termination remains unknown. Field data exclude hyperthermia as the sole proximate explanation (Hetem *et al.*, 2013), but PCr availability, Pi and proton handling, glucose and glycogen dynamics, oxygen delivery, neuromuscular factors and behavioural decisions all remain plausible contributors.

### Recovery is asynchronous and process-specific

Recovery begins immediately after the chase, but different processes recover on different timescales. In humans, muscle PCr recovered to ~80% of its resting concentration within 6 minutes after repeated maximal sprints, while muscle pH remained depressed (Mendez-Villanueva *et al.*, 2012). Lactate and protons are rapidly exported from contracting muscle, but muscle-to-blood lactate exchange and whole-body lactate removal follow distinct recovery kinetics (Chatel *et al.*, 2016; Brooks, 2018). Systemic acid–base and electrolyte normalization, glycogen replenishment and downstream metabolite processing can extend over hours or longer. Under favourable nutritional conditions, human muscle glycogen can be substantially restored within ~24 hours (Ørtenblad *et al.*, 2013; Hargreaves and Spriet, 2020), whereas glycogen remained depleted 3 days after a 15-minute chase in blesbok and showed supercompensation by day 16 (Kohn *et al.*, 2025). Digestion and post-prandial metabolism also unfold over hours.

Exercise recovery does not depend on immediate feeding. If a chase ends without a kill, PCr resynthesis begins as soon as oxidative ATP production can support the creatine kinase reaction, and some muscle glycogen can be restored during fasting from endogenous carbon sources such as lactate (Fournier *et al.*, 2004). The proposed overlap therefore arises specifically after a successful hunt, when feeding begins while one or more slower recovery processes may still be active.

The model therefore focuses on process-specific overlap rather than a binary recovered/unrecovered state. After a successful hunt, feeding may begin after PCr has largely recovered but while muscle pH, glycogen stores, systemic acid–base balance or downstream metabolite processing remain displaced. Lactate may have left the muscle while its oxidation or conversion elsewhere continues. The variables that overlap, and their timing, may differ among hunts, environments and individuals.

These recovery curves have not yet been measured in cheetahs and may be shaped by species-specific adaptations. The model would be weakened if the variables predicted to overlap have already returned towards their pre-event trajectories before feeding begins, or if changing the hunt-to-feed interval does not alter the combined exercise and post-prandial response.

### Feeding ecology as a second metabolic load

After prey capture and restraint, feeding introduces the second major metabolic load in the proposed sequence. Cheetahs consume substantial carcass meals, often under pressure from larger competitors, which concentrates digestive and metabolic work into a relatively short period (Caro, 1994; Marker *et al.*, 2003).

Gastric digestion produces a large acid–base flux. Hydrochloric acid (HCl) secretion into the stomach is matched by bicarbonate release into the circulation, creating the post-prandial alkaline tide (Niv and Fraser, 2002; Gennari and Weise, 2008). Pancreatic and biliary bicarbonate is then secreted into the intestine to neutralize gastric acid and support digestion (Hall, 2020). Chloride, sodium and potassium handling are closely linked to these processes and to the renal response that follows.

The meal also creates a major substrate load and has its own energetic cost. Digestion and absorption require muscular, secretory and transport work. Early post-prandial metabolism includes the breakdown, transport and oxidation of dietary substrates; as absorbed nutrients become available, protein synthesis, glycogen replenishment and other anabolic processes increase (Hall, 2020). Amino acid catabolism increases urea production and generates organic-acid intermediates. Lipid digestion requires bile acid secretion and enterohepatic recycling. Bile acids also regulate metabolism, inflammation and mitochondrial function through the farnesoid X receptor and G protein-coupled bile acid receptor 1 (Hofmann, 2009; Chiang, 2013).

Glycine conjugation helps clear aromatic and other carboxylic acids. The reaction occurs in mitochondria through acyl-CoA:glycine N-acyltransferase and is active in both hepatic and renal tissues (Badenhorst *et al.*, 2014). Limited glucuronidation in felids increases the importance of alternative conjugation pathways (Court, 2013; Trepanier, 2013). The abundance of glycine-conjugated metabolites in cheetah urine is consistent with substantial flux through this system (Tordiffe *et al.*, 2017).

The model proposes that these post-prandial processes can overlap with the slower components of exercise recovery. Two intervals are important. The hunt-to-feed interval determines the immediate overlap between sprint recovery and digestion. The inter-meal interval determines whether slower post-prandial changes return towards baseline before the next major nutrient input. Feeding is therefore not a passive recovery event, but a second physiological challenge whose effect depends on its timing.

The timing of first ingestion, the rate and amount consumed in the first feeding bout, and the post-prandial acid–base response have not been quantified against recovery variables in free-ranging cheetahs. The digestive mechanisms above are established mainly in other mammals and felids. Their interaction with cheetah sprint recovery is the central untested step in the hypothesis. This sequence is summarized in Fig. 1.

**Figure 1:**
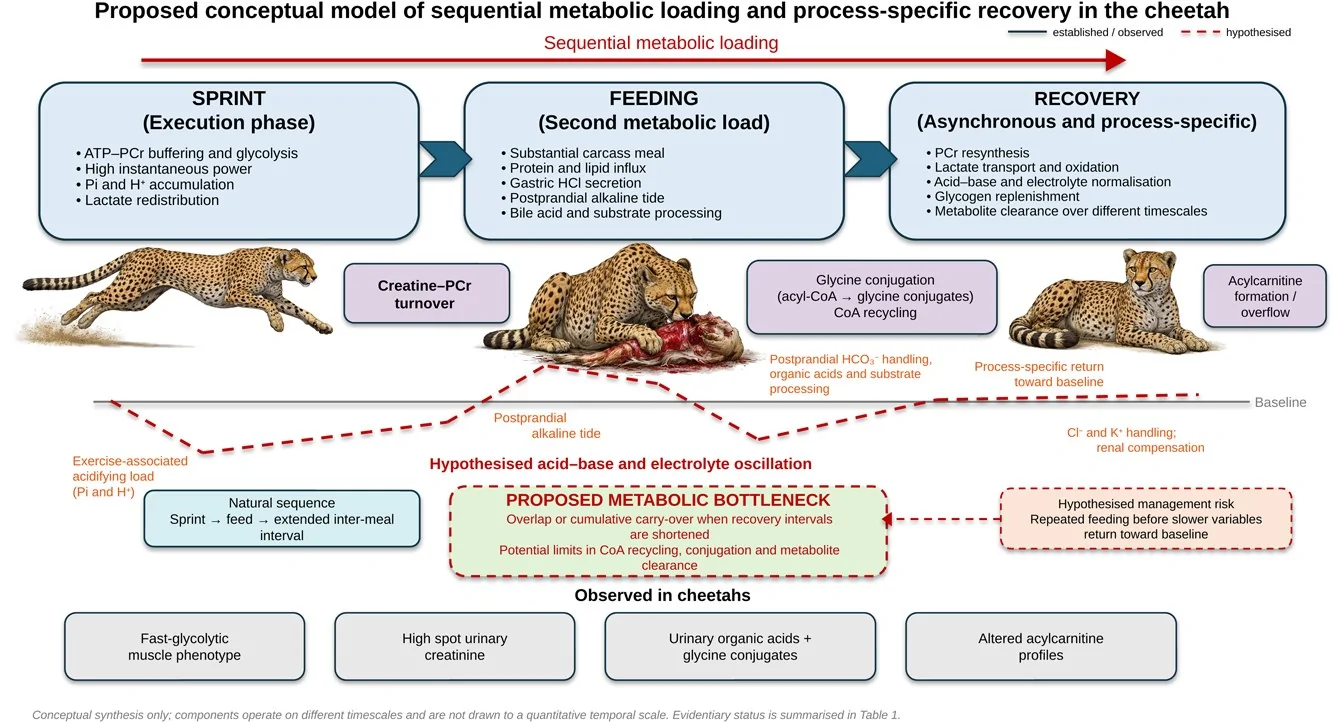
Proposed conceptual model of sequential metabolic loading and process-specific recovery in the cheetah. The figure presents the central hypothesis: the same high-rate energy systems that enable sprinting create a metabolic cost, and feeding after a successful hunt may add a second, overlapping load. It is not drawn to a quantitative timescale. Solid arrows denote the observed behavioural sequence or established relationships; dashed arrows denote hypothesized causal links or unmeasured physiological trajectories. Recovery begins when sprinting ceases, but individual processes are expected to resolve asynchronously. Sprinting is proposed to alter PCr availability, Pi and proton balance, lactate distribution and substrate use. Feeding then imposes a second metabolic load through gastric HCl secretion and the systemic alkaline tide, pancreatic and biliary bicarbonate flux, bile acid signalling, and amino acid and lipid processing. The model predicts that one or more slower exercise-recovery processes may overlap with this post-prandial load. A metabolic bottleneck is hypothesized only if shortened hunt-to-feed or inter-meal intervals produce measurable carry-over, such as prolonged acid–base or electrolyte disturbance, delayed metabolite clearance, or cumulative organic-acid and acylcarnitine changes. The observed cheetah findings shown at the bottom of the figure motivate the model but do not establish these causal links; their evidentiary status and critical tests are summarized in Table 1.

### Biochemical signatures of a high-flux system

Against this framework, the available biochemical observations can be considered as signs of a system organized for rapid energy flux.

The PCr system is a rapid temporal buffer for ATP. Creatine kinase transfers phosphate between PCr and ADP, allowing ATP concentration to be maintained briefly when demand exceeds oxidative supply (Wallimann *et al.*, 2011; Hargreaves and Spriet, 2020). The cheetah’s glycolytic muscle phenotype is consistent with strong reliance on this high-rate buffer at the start of a sprint (Kohn *et al.*, 2024).

Published urinary data provide an additional clue. In 41 apparently healthy captive cheetahs, spot urinary creatinine concentrations ranged from 12.9 to 115.4 mmol L^−1^, with a mean of 48.64 mmol L^−1^; mean urine specific gravity was 1.054 (Tordiffe *et al.*, 2017). Creatinine is formed by spontaneous, irreversible conversion of creatine and PCr, and daily production is related to the size of the body’s creatine pool (Casey and Greenhaff, 2000; Brosnan and Brosnan, 2007; Ostojic, 2021).

These concentrations are compatible with a large or active creatine–PCr pool and therefore fit the proposed high-flux phenotype. If such a pool is present, its maintenance would depend on dietary creatine and endogenous synthesis from glycine, arginine and methionine, linking sprint energetics to post-prandial amino acid metabolism.

Spot urinary creatinine concentration cannot demonstrate acute PCr turnover. It is also influenced by urine dilution, muscle mass, diet, renal handling, sampling conditions and age. The hypothesis therefore requires direct measurement of muscle total creatine and PCr, exercise-related depletion and resynthesis kinetics, and preferably absolute creatinine excretion under standardized conditions.

### Glycine, acylcarnitines, and metabolic fragility

Glycine sits at the intersection of several pathways that may become important during sequential loading. It is required for creatine synthesis, glutathione production and conjugation of organic acids, linking energy buffering, redox defence and metabolite clearance.

During glycine conjugation, aromatic and other carboxylic acids are first activated to acyl-CoA intermediates and then conjugated by acyl-CoA:glycine N-acyltransferase (Badenhorst *et al.*, 2014). This reaction promotes excretion of the acid and releases coenzyme A (CoA) for reuse. High substrate delivery can therefore increase both glycine demand and the need to maintain free CoA.

Urinary organic-acid profiles in cheetahs contain numerous glycine conjugates and other metabolites associated with amino acid, carbohydrate and fatty-acid metabolism (Tordiffe *et al.*, 2017). Acylcarnitines provide a parallel route for handling acyl groups. Their formation supports mitochondrial transport and can also export excess acyl intermediates when substrate delivery exceeds complete oxidative processing. Altered acylcarnitine profiles in cheetahs are consistent with this type of metabolic overflow (Tordiffe, 2017).

The hypothesis links these findings through a common bottleneck. Sprint recovery, digestion and amino acid catabolism all draw on mitochondrial processing, CoA availability and metabolite-clearance pathways. Glycine is required at the same time for conjugation, creatine synthesis and antioxidant defence. If demand exceeds pathway capacity, organic acids, acylcarnitines or other intermediates should accumulate and clear more slowly.

The kidney contributes to this system through acid–base and electrolyte regulation, amino acid handling and excretion of conjugated metabolites. Repeated or overlapping loads could therefore increase renal work even when primary renal disease is absent.

The cheetah data demonstrate metabolite patterns compatible with this model, but they do not show that glycine, CoA recycling or mitochondrial processing is actually limiting. That requires time-resolved measurements across exercise, feeding and recovery, together with direct tests of dietary glycine availability and conjugation capacity.

### Clinical and conservation implications

The model offers a possible physiological explanation for several recurring findings in cheetahs under human care. These include variable gastrointestinal function, inconsistent faecal quality, episodic diarrhoea, altered intermediary metabolites and fluctuations in acid–base or electrolyte measurements. Such findings may reflect a system in which digestive and metabolic inputs are not always separated by enough time for slower variables to return towards baseline.

Feeding management is therefore a plausible point of intervention. Meal frequency, meal composition and the interval after exercise may alter gastric acid secretion, bile acid exposure, substrate delivery and the time available for metabolite clearance. Diets rich in collagen and other carcass components may also affect glycine supply and colonic fermentation compared with diets based mainly on muscle meat.

For conservation breeding and long-term managed care, the practical prediction is simple: husbandry should preserve adequate intervals for recovery and avoid creating repeated physiological inputs that produce cumulative carry-over. The relevant interval may differ among variables and should be measured rather than assumed.

These clinical and management links remain hypotheses. Gastrointestinal disease in captive cheetahs is likely to be multifactorial, with possible contributions from diet composition, food handling, stress, activity, microbiota and institutional husbandry. Feeding frequency should therefore be tested as one component of a broader causal model, not treated as an established sole cause.

### Incomplete resolution, carry-over and metabolic instability

Within the proposed model, metabolic instability develops when one or more disturbances persist into the next physiological input. The model does not require every variable to return fully to its pre-event value before the next input. The important feature is carry-over: a variable remains displaced long enough to change the response to feeding, another exercise bout or the next meal.

Carry-over could involve intracellular proton or inorganic-phosphate handling, systemic acid–base and electrolyte regulation, glycogen restoration, lactate oxidation, bile acid cycling, glycine-dependent conjugation, acylcarnitine clearance or renal excretion. Repeated overlap would shift the system from separate, reversible responses towards a persistently perturbed state.

Under managed conditions, short inter-meal intervals, altered diet composition or other environmental pressures could expose this narrow recovery margin. Disease would then emerge from the cumulative interaction of several incomplete processes rather than failure of a single pathway.

No direct evidence currently shows harmful carry-over after normal hunts in free-ranging cheetahs. Behavioural delays, exceptional buffering, rapid metabolite transport or other species-specific adaptations may prevent this. The model is therefore most useful as a way to identify and test those adaptations and to determine when altered management exposes constraints that are normally well contained.

### Evidence and testable predictions

The model can be tested by establishing time-resolved recovery profiles after standardized exercise and then determining whether the timing of feeding changes them (Table 1). Serial blood, urine and, where feasible, muscle measurements should identify which variables recover within minutes and which remain displaced for hours or longer. The key experiments are to vary the hunt-to-feed interval and the interval between meals while holding exercise, nutrient intake and diet composition constant. The model predicts that shorter intervals will prolong or amplify acid–base, electrolyte, glucose, bile acid and metabolomic disturbances, including changes in organic acids, glycine conjugates, acylcarnitines and creatine-related measures. It would be weakened if relevant variables return towards baseline before feeding, if interval manipulation has no measurable effect, or if the proposed biochemical bottlenecks do not vary with metabolic load. Management recommendations should follow only from these interval-dependent results.

## Conclusion

The central hypothesis is straightforward: the adaptations that allow cheetahs to generate ATP at exceptional rates also create a metabolic cost that must be resolved after a chase. Their feeding ecology may amplify this cost, because a large meal can impose a second physiological load while slower recovery processes remain active. The model would be weakened if the variables predicted to overlap have already returned towards their pre-event trajectories before feeding begins, if interval manipulation produces no measurable change, or if the proposed biochemical bottlenecks do not vary with metabolic load.

## Data Availability

No data were generated or analysed during this work.
